# Atmospheric Spray Freeze Drying of Sugar Solution With Phage D29

**DOI:** 10.3389/fmicb.2019.00488

**Published:** 2019-03-20

**Authors:** Alvin Ly, Nicholas B. Carrigy, Hui Wang, Melissa Harrison, Dominic Sauvageau, Andrew R. Martin, Reinhard Vehring, Warren H. Finlay

**Affiliations:** Department of Engineering, University of Alberta, Edmonton, AB, Canada

**Keywords:** phage processing, freeze-dry, alternative process, powder production, *Mycobacterium tuberculosis*

## Abstract

Therapeutic bacteriophages offer a potential alternative approach in the treatment of drug resistant bacteria. In the present study, we examine the ability of atmospheric spray freeze-drying (ASFD) to process bacteriophage D29 into a solid dry formulation. Bacteriophage D29 is of particular interest due to its ability to infect *Mycobacterium tuberculosis*. A sugar solution containing bacteriophage D29 was sprayed and instantly frozen in a cold chamber. Cold drying gas was then passed through the chamber at a high flow rate and atmospheric pressure. Convective transport combined with the low temperature of the drying gas results in sublimation of ice, yielding a free-flowing, porous powder. The bacteriophages were atmospheric spray freeze-dried in solutions with varying concentrations of trehalose and mannitol. A solution of trehalose and mannitol at a mass ratio of 7:3 and a total mass concentration of 100 mg/mL led to powder with 4.9 ± 0.1% moisture content and an acceptable titer reduction of ∼0.6 logs. In comparison, a pure trehalose solution and a 1:1 ratio of trehalose and mannitol both had titer reductions of >1.5 logs. Spectroscopic analysis showed that trehalose in the powder was amorphous while mannitol completely crystallized during the drying process, both of which are desirable for preserving phage viability and storage in powders. The results highlight the potential for using ASFD as an alternative process in preserving biopharmaceutical products.

## Introduction

With the recent emergence of multiple drug resistant bacteria, interest in therapeutic bacteriophage (phage) applications has increased. Phages present an alternative resource in combating bacteria-related diseases (Kutateladze and Adamia, 2010). Phages are viruses that infect bacteria and can have a narrow host range of target bacteria (Hatful and Vehring, 2016; Dewangan et al., 2017). This narrow host range results in little to no off target effects, unlike what is seen with conventional antibiotics which often damage microbiota (Loc-Carrillo and Abedon, 2011; Dewangan et al., 2017). Phages and antibiotics use different mechanisms in killing bacteria, so antibiotic-resistant bacteria can remain sensitive to killing by phages (Loc-Carrillo and Abedon, 2011). Although bacteria can develop resistance to phages, phages can mutate to overcome phage-resistance in the bacteria and phage cocktails may be useful for limiting resistance to one type of phage (Kutateladze and Adamia, 2010; Loc-Carrillo and Abedon, 2011; Dewangan et al., 2017).

There are many phages with varying structures, one of which consists of a polyhedral capsid containing genetic material and a tail used to bind to the bacteria and inject the genetic material (Hatful and Vehring, 2016; Dewangan et al., 2017). Although not all phages can be used as therapeutic agents, lytic phages tend to be the most useful, due to their exponential growth and effectiveness in eradicating the host bacteria (Kutateladze and Adamia, 2010). Unlike temperate phages, lytic phages have not been shown to transfer DNA between host bacteria, therefore eliminating the risk of spreading antibiotic or phage resistance genes (Loc-Carrillo and Abedon, 2011). Lytic phages operate by binding to and infecting host bacteria, multiplying within the host bacteria, and killing the host bacteria by rupturing the cell wall before releasing progeny and spreading to neighboring bacteria (Dewangan et al., 2017).

One species of bacteria that exhibits multiple drug resistance is *Mycobacterium tuberculosis*, which caused an estimated 480,000 cases of multi drug resistant tuberculosis in 2014, only half of which were successfully treated (World Health Organization, 2018a). There have also been cases of totally drug-resistant tuberculosis that can no longer be treated with any available antibiotics; therefore, phage therapy is an interesting alternative treatment (Udwadia, 2016). While each pathogen is susceptible to different strains of phages, for tuberculosis, *Mycobacterium* virus D29 is one of the most effective phages against this host (Guo and Ao, 2012; Hatful and Vehring, 2016; Lapenkova et al., 2018). Phage D29 are not only effective against tuberculosis but are also safe to culture since they can be grown on *Mycobacterium smegmatis* rather than on *M. tuberculosis* (Hatful and Vehring, 2016). Liu et al. (2016) showed that inhalation of phage D29 resulted in a higher dose of phage D29 reaching and staying in the lungs compared to injection in mice. Carrigy et al. (2017) compared the titer reduction in different inhalation devices for the delivery of phage D29 and found that titer reduction was highly dependent on the inhalation device, with a vibrating mesh nebulizer achieving the lowest titer reduction.

In 2016, approximately 70% of new tuberculosis cases occurred in either Asia or Africa (World Health Organization, 2018b) in countries where a continuous cold-chain cannot always be guaranteed. In order to better utilize the potential of phage therapy in these regions, the development of phages in a dry solid form would be useful since phages, which are often primarily composed of protein, are more susceptible to deactivation in liquid form as compared to a dry solid state (Wang, 2000). Therefore, it can be expected that a suitable dry dosage form would allow for higher storage temperatures. The most common method of preserving proteins in a dry solid state is tray lyophilization (Wang, 2000). Zhang et al. (2018) successfully freeze-dried an M13KE model phage and showed that the phages had higher survivability as a dry powder compared to liquid formulations for a storage period of 60 days; the dry powders were stored at room temperature whereas the liquid formulations were stored at 4°C. Although there has been success in preserving phages with tray lyophilization, this technique is limited by cryoprotectant solubility and long processing times (Leuenberger, 2002; Ishwarya et al., 2015).

Spray drying is an alternative approach to preserving phages in a dry solid form. The drying process involves spraying a formulation into a chamber with concurrent flow that rapidly evaporates the solvent in the particles. Although spray drying presents an attractive alternative due to its faster processing time and ability to produce fine particles for inhalation, different phage strains are sensitive to different environmental and process conditions with the result that some phage species may have reduced viability after spray drying (Vandenheuvel et al., 2013). Another alternative approach in preserving phages in a solid form is by using spray-freeze drying. Spray freeze-drying involves spraying a formulation into a cold medium and drying under vacuum and temperature conditions similar to lyophilization. The freezing step in spray freeze-drying preserves the spherical shape of the droplets and reduces degradation due to phase separation, which is common in lyophilization (Heller et al., 1999). Spray freeze-drying has been shown to produce powders with particles larger and more porous than spray drying (Maa et al., 1999). Proteins that are spray freeze-dried are sensitive to the drying process and stabilization characteristics are improved with the excipients included and the intermediate drying steps (Sonner et al., 2002; Maa et al., 2004). Leung et al. (2016) examined the feasibility of both spray drying and spray freeze-drying of phages and the aerosolization characteristics of the resulting dry powders. Both processes had similar overall titer reduction; in the case of spray freeze-drying, titer reduction was caused mainly by the atomization step. In some cases, spray freeze-drying forms more aggregates compared to spray drying and is not feasible for all pharmaceutical ingredients (Nguyen et al., 2004). The frozen powders in spray freeze-drying are dried under vacuum pressure. As a result, this process suffers from long drying times similar to tray lyophilization (Shoyele and Cawthorne, 2006).

Atmospheric spray freeze-drying (ASFD) is a relatively new technique in protein preservation that is an alternative to the above noted processes. Meryman (1959) was the first to show that freeze-drying is based on the relative vapor pressure at the surface of the frozen object compared to the ambient pressure, rather than the absolute vapor pressure, concluding that it is possible to freeze-dry without the use of a vacuum environment. ASFD has improved mass and heat transfer rates, therefore decreasing drying time, giving high and homogenous quality to the dried product, and producing free-flowing powder with larger surface area and increased solubility (Mumenthaler and Leuenberger, 1991; Ishwarya et al., 2015). Malecki et al. (1970) first used ASFD for liquid formulation in food products, where the technology was used to dry liquid food and juices. ASFD is also capable of freeze-drying thermosensitive pharmaceutical products that are difficult to properly dry in tray lyophilization (Leuenberger et al., 2006; Wang et al., 2006). Leuenberger (2002) developed a system to freeze-dry liquid pharmaceutical products using ASFD with a fluidized bed in a single freeze-drying chamber. Wang et al. (2006) developed an ASFD system without a fluidized bed, leading to decreased drying time and low degradation of both proteins and bacteria formulations. Sebastiao et al. (2017) developed a numerical model to determine the optimal drying temperature and time for ASFD. The numerical model was compared to experimental drying gas and temperature data, which can be used to predict optimal drying cycles for different active pharmaceutical ingredients, droplet characteristics, and a scaled up ASFD apparatus. The present paper explores the possibility of freeze-drying phage D29 with ASFD.

## Materials and Methods

### Atmospheric Spray Freeze-Drying

**FIGURE 1:**
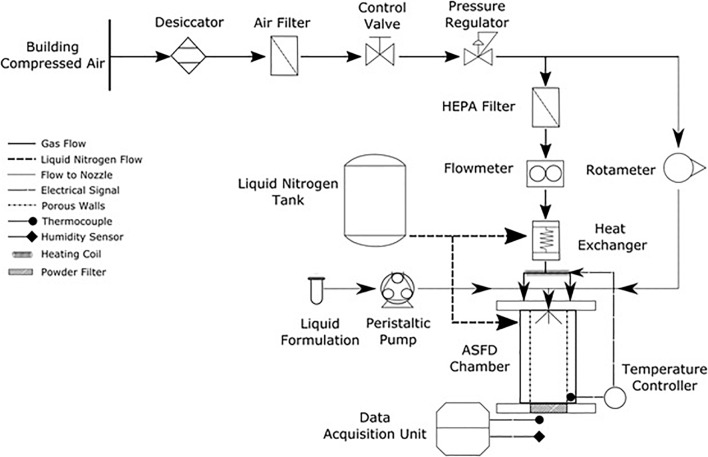
Schematic of the developed ASFD apparatus.

An ASFD apparatus that improves upon the design given by Wang et al. (2006) was developed. A schematic of the apparatus is shown in Figure 1. The drying gas is supplied by a building compressed air line through a ½-inch polycarbonate tube. The compressed air first enters a desiccator (Arrow Pneumatics D10-04, Broadview, IL, United States) containing silica gel beads to dry the air prior to its exit through a 40-micron filter. The dry air is further cleaned by an additional 0.5-micron filter (Wilkerson AF1-04-S00, Richland, MI, United States). Pressure of the dry gas is reduced after the filter by a compressed air regulator (Norgren R73G-2AK-RMN, BI, United Kingdom). The dried and filtered compressed air splits into two lines. One line supplies atomizing gas to the twin-fluid nozzle with its flow rate controlled and measured by a rotameter (Cole Parmer, MTN, Canada). Drying gas supplied by the other line is regulated by a control valve (Swagelok B-1RF4, OH, United States) and then enters a HEPA filter that is connected to a TSI flow meter (4043, Shoreview, MN, United States). A coiled ½-inch copper tube is used to supply the drying gas through a heat exchanger that is filled with liquid nitrogen from a supply tank (Praxair, Edmonton, AB, Canada) to chill the drying gas. The heat exchanger is an upright sealed steel cylinder chamber. A heating rope (OMEGALUX FR-060, Laval, QC, Canada) is wrapped around the copper tube at the exiting end of heat exchanger to heat the drying gas to designated operating temperatures.

The ASFD chamber consists of five main components: an aluminum top lid, a twin fluid nozzle (Spray System Co. 1/8 JJCO, Wheaton, IL, United States), a porous metal cylinder (Mott, Farmington, CT, United States), an outer chamber, and a porous filter disk (Applied Porous Technologies Inc., Tariffville, CT, United States). The top lid has vent bores, a connector for liquid nitrogen filling, and connectors for copper tubing. The drying gas enters through the top lid and flows into the aluminum outer chamber. The porous cylinder is contained within the outer chamber and creates a gas buffer that prevents the sprayed particles from contacting the porous cylinder while directing the flow of particles to the filter disk. The twin fluid nozzle is connected to the top lid. The nozzle has a gas supply from the compressed air line while the liquid is supplied by a peristaltic pump (Chem-Tech CTPA4LSA, Punta Gorda, FL, United States). The frozen powder is collected on the filter disk, which sits on an aluminum piece that is attached to the bottom of the ASFD chamber. The filter disk is made of chemical and corrosion-resistant 316L Stainless Steel with a pore size of 20 μm.

A Type T thermocouple, which is connected to a feedback system (OMEGA CN742, Laval, QC, Canada) that controls the heating rope, was placed at the bottom of the ASFD chamber near the powder. Using the temperature near the powder, the heating rope warms the cold drying gas to the desired operating temperature. A similar thermocouple is placed at the exit of the ASFD chamber to measure the temperature of the outlet gas, which was recorded using a temperature logger (National Instrument USB-TC01, Austin, TX, United States). The humidity of the outlet gas is measured with a humidity probe (Vaisala HMP75B, Woburn, MA, United States) connected to a hand-held indicator (Vaisala MI70, Woburn, MA, United States).

### Operating Procedure

The drying process was split into two main steps. The first step was the spray-freezing step. The ASFD chamber was first cooled with liquid nitrogen and dry air. Liquid nitrogen was pumped into the chamber while filtered and desiccated air flowed through the system. The chamber was cooled and upon stabilizing at -130°C, the liquid nitrogen was shut off and the phage solution was pumped through the twin fluid nozzle. The liquid solution was pumped at 20 mL/min while the compressed air flowed through the nozzle at 10 L/min. At the end of spraying the ASFD chamber had warmed to -80°C.

**FIGURE 2:**
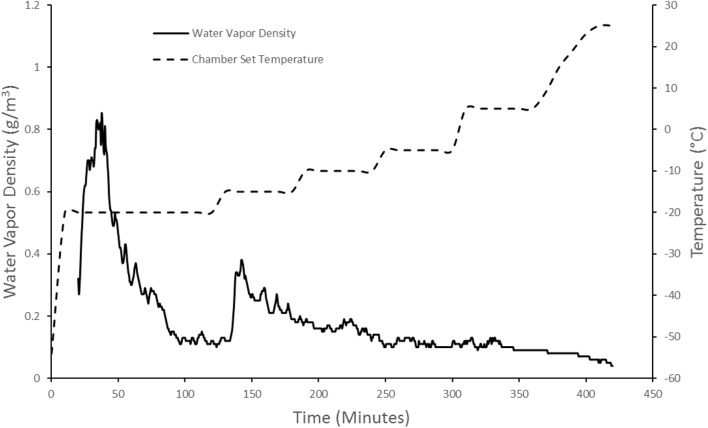
ASFD process development that correlates chamber set temperature and water vapor density of outlet gas. The solid black line corresponds to the water vapor density graph. The dashed line corresponds to the chamber set temperature. The water vapor density measurement starts 20 min into the run due to the humidity probe’s lower operating limit of –50°C.

The second step in ASFD was atmospheric drying. This began by gradually increasing the temperature of the chamber from -80 to -20°C. The drying gas temperature was regulated by the rope heater and the volume of liquid nitrogen in the heat exchanger. The chamber temperature was kept at -20°C for 2 h, and then held constant for 1 h each at incrementally higher temperatures. The temperature was eventually brought up to 25°C by using the heating rope to warm the drying gas. The initial temperature hold of -20°C was chosen to promote crystallization of mannitol (Mehta et al., 2013). The moisture of the freeze concentrate could not be measured throughout ASFD so a trial and error process with the outlet gas moisture was used to determine the drying gas temperature. Since the water vapor density of the outlet gas was independent of the gas temperature, the vapor density was used to determine the chamber temperature throughout the drying process instead of the relative humidity. The water vapor density was used as a qualitative indicator to determine when the mass transfer of water from the powder has slowed or stopped completely. When the vapor density of the outlet gas decreased and reached a plateau, the temperature of the drying gas was increased. In order to lower the moisture content of the phage powder, the drying time was increased by 1 h in the second set of phage powders. Figure 2 shows the water vapor density of the outlet gas and the drying chamber set temperature for a typical run.

The powder sample was collected by removing the filter at the end of the ASFD process. The samples were stored in plastic vials (Fisher-Scientific Eppendorf Tubes, Ottawa, ON, Canada) inside a drybox at 25°C. To study the effect of storage temperature, a portion of the powder samples were also stored in a drybox in the fridge at 4°C.

### Formulation Preparation

ASFD was used to produce powders with phage D29 using three formulations with varying trehalose and mannitol mass ratios, for a total mass concentration of 100 mg/mL. Table 1 shows the three formulations and two duplicate samples used to freeze-dry phage D29. For the A2 and B2 powders, the ASFD process was increased from a 6-h drying process (for powders A, B, C) to a 7-h drying process. Phage D29 was amplified on the host *Mycobacterium smegmatis* strain mc^2^155 with a solid media. The phage buffer was poured onto plaques of host bacterium and incubated overnight at 4°C and filtered through a 0.22 μm filter (Carrigy et al., 2017). In the current proof-of-concept stage, only basic purification is required, whereas more refined procedures would have to be developed for the manufacturing stage of therapeutic phages (Pirnay et al., 2015). The liquid formulation contained either trehalose or a mixture of trehalose and D-mannitol, with the addition of D29 phages to a concentration ranging from 1.5 to 5.2 × 10^11^ pfu/mL. The D-(+)-trehalose dihydrate (T9531, St. Louis, MO, United States) and D-mannitol (M4125, St. Louis, MO, United States) were both purchased from Sigma-Aldrich. Trehalose and D-mannitol were dissolved in 10 mL of deionized water at the proper mass ratio and concentrations. The solution was then filtered with a 0.22-μm pore size filter. A micropipette was used to add 50 μL of D29 phage at a concentration ranging from 1.5 to 5.2 × 10^11^ pfu/mL to the solution that was then vortexed; 500 μL of the phage solution was separated for titer measurement of the liquid formulation while the remaining liquid solution was freeze-dried with the ASFD apparatus.

**Table 1:** Feed solution composition for atmospheric spray freeze-drying. The total mass concentration of trehalose and mannitol was 100 mg/mL for each case.

ASFD feed solution	Mass ratio (% w/w)		Volume (mL)	Process time (min.)
			
	Trehalose	Mannitol		
A	100	0	10	360
B	70	30	10	360
C	50	50	10	360
A2	100	0	10	420
B2	70	30	10	420


### Moisture Measurement

The residual moisture of the freeze-dried powder was measured gravimetrically by comparing the weight of the sample before and after oven drying. The mass of the ASFD powders was measured on a glass dish and placed into an isotherm oven (Fisher-Scientific Isotemp 280A, Hampton, NH, United States). A 100 mg sample of each powder was heated to 100°C at 3.0 kPa absolute pressure for 8 h and slowly cooled in the oven overnight. The vacuum oven was periodically vented and pumped to ensure that the air around the sample was dry.

### Titer Measurement

Plaque assays on the surrogate host *M. smegmatis* mc^2^155 were used to measure the titer of the phages in plaques-forming units per mL (pfu/mL). The prepared samples were in freeze-dried solid form and were resuspended in ∼1 mL of phage buffer prior to plaque assay and the subsequent titer measurement. The full plate titer method was used since plaques were too large to use the spot assay method. Details of the plaque assay method are given elsewhere (Bioscience, 2018).

### Scanning Electron Microscope

Images of the resulting powders were taken using a field emission scanning electron microscope (SEM) (ZEISS Sigma FE-SEM, Oberkochen, Germany). The images were taken with the immersion lens detector with a working distance of 6.0 mm and an accelerating voltage of 3.0 kV. Each sample was coated with a 10 nm layer of gold prior to imaging using a Denton vacuum gold sputter unit (Desk 2, Moorestown, NJ, United States).

### Raman Spectroscopy

A custom-designed dispersive Raman spectrometer was used for solid phase analysis of the dried powders. A 671 nm diode-pumped laser (Ventus 671, Laser Quantum, United Kingdom) with a maximum output power of 500 mW was used as the excitation source. The powders were loaded into a conical cavity with a volume of 0.2 μL in an aluminum sample holder and kept under a nitrogen atmosphere during the measurement. All measurements were conducted at a temperature of 23 ± 1°C and a relative humidity of less than 3%. Pure trehalose and its spray-dried form were measured, respectively, to obtain the spectra for crystalline and amorphous trehalose. Three polymorphic forms of mannitol, α, β, and δ, were also produced and measured for their Raman reference spectra. D-mannitol was tested directly as received and used as the reference for β-mannitol. The other two forms of polymorphs were prepared using a slow solvent evaporation method (Wang et al., 2014). Briefly, α-mannitol was obtained by adding D-mannitol to a mixed solvent of acetone, ethanol, and water in the volume ratio of 5:5:2 at 50°C during electromagnetic stirring and then cooling the solution down to room temperature to allow crystal precipitation. The δ-form of mannitol was achieved by drying the aqueous solution of D-mannitol at room temperature in a vacuum desiccator.

A deconvolution process (Wang et al., 2017) was used to determine the contributions of each component in multi-component systems according to Eqn. (1)

SRe⁡s=SRaw−[B+Σ(Ii±ei)Si,N]

In which *S_Res_* is the residual spectrum after subtracting all components from the raw spectrum of the multi-component mixture,*S_Raw_*, and *B* is the background signal. The normalized reference spectrum,*S_i,N_*, is used as the spectral intensity unit for the corresponding pure component and the spectral contribution of the component to the raw spectrum of the mixture is *I_i_S_i,N_*. The deconvolution approach was performed manually by iteratively adjusting the intensity factor, *I_i_*, to minimize the residuals for each component. Uncertainty of the intensity factor for each component, ± *e_i_*, is a range within which the corresponding component is no longer distinguishable between under- and over-subtraction. The intensity factors for each component are directly related to their corresponding mass fractions.

## Results

### Titer Measurement

**FIGURE 3:**
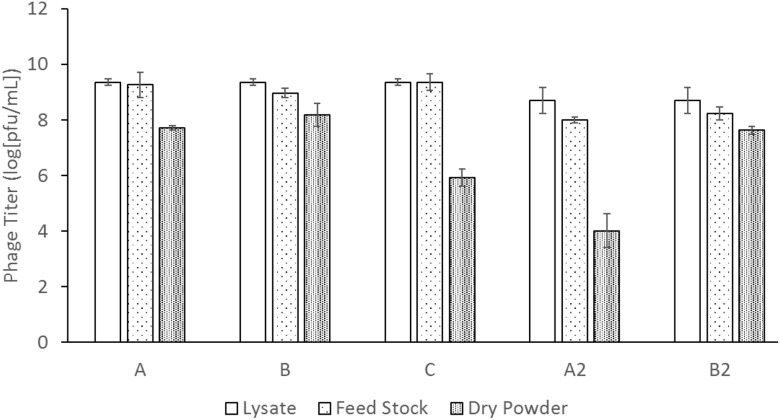
Phage titer measurement of the lysate, liquid solution, and dry powder for each solution. The labels A, B, C, A2, and B2 indicate different feed solution compositions and process times defined in Table 1. Three repeat titer measurements of each data point were used to determine the error bars of the phage titer.

The absolute titers of the phage measured for (a) the phage lysate, (b) the feed stock, i.e., the lysate mixed with sugar formulation, and (c) the final freeze-dried powder of phages is shown in Figure 3. Trehalose and mannitol at a 1:1 mass ratio (powder C) had an unacceptably large titer reduction of ∼3 logs, from the feed stock to the dried powder; therefore, feed stock for powder C was not used again. This result is consistent with previous phage research where lower mass ratios of trehalose had lower phage survivability (Leung et al., 2017). In both titer measurements of powder B and B2 – with a trehalose to mannitol mass ratio of 7:3, the ASFD process had a consistent titer reduction from the feed stock to the freeze-dried powder of ∼0.8 and ∼0.6 logs, respectively. The pure trehalose powder had high titer reductions of ∼1.5 and ∼4.0 logs, for powders A and A2, respectively.

### Moisture Content

**FIGURE 4:**
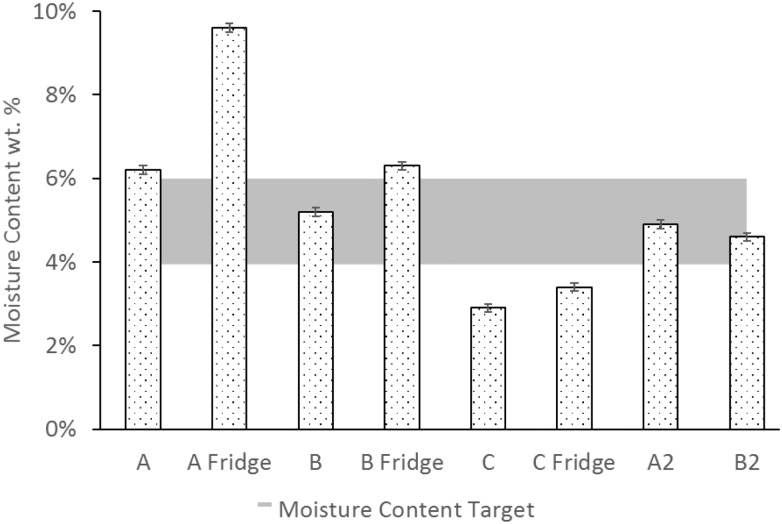
Moisture content of each powder stored in a drybox either at room temperature (25°C) or in the fridge (4°C). Shaded area (4–6%) represents the ideal powder moisture content for phages. The labels A, B, C, A2, and B2 indicate different feed solution compositions and process times defined in Table 1.

The moisture contents of the ASFD powders are shown in Figure 4. The first batch (A) had two different storage conditions in order to determine the best short-term storage environment. One set of powder was stored in a drybox at room temperature (25°C), while another set of powder was stored in a refrigerator at 4°C. Powder A had a moisture content of 6.2 ± 0.1% w/w when stored in a drybox at room temperature and a moisture content of 9.6 ± 0.1% w/w when stored in a drybox for one week in the fridge. Powder B had a moisture content of 5.2 ± 0.1% w/w when stored in the drybox and a moisture content of 6.2 ± 0.1% w/w when stored in the fridge. Based on the differences in moisture content and negligible effects on titer measurement, the duplicate phage powders (A2, B2) were only stored in a drybox at room temperature. The additional hour of drying time used with the duplicate runs (A2, B2) decreased the moisture content to 4.6 ± 0.1% w/w and 4.9 ± 0.1% w/w for powders A2 and B2, respectively.

### Solid Phase Properties

**FIGURE 5:**
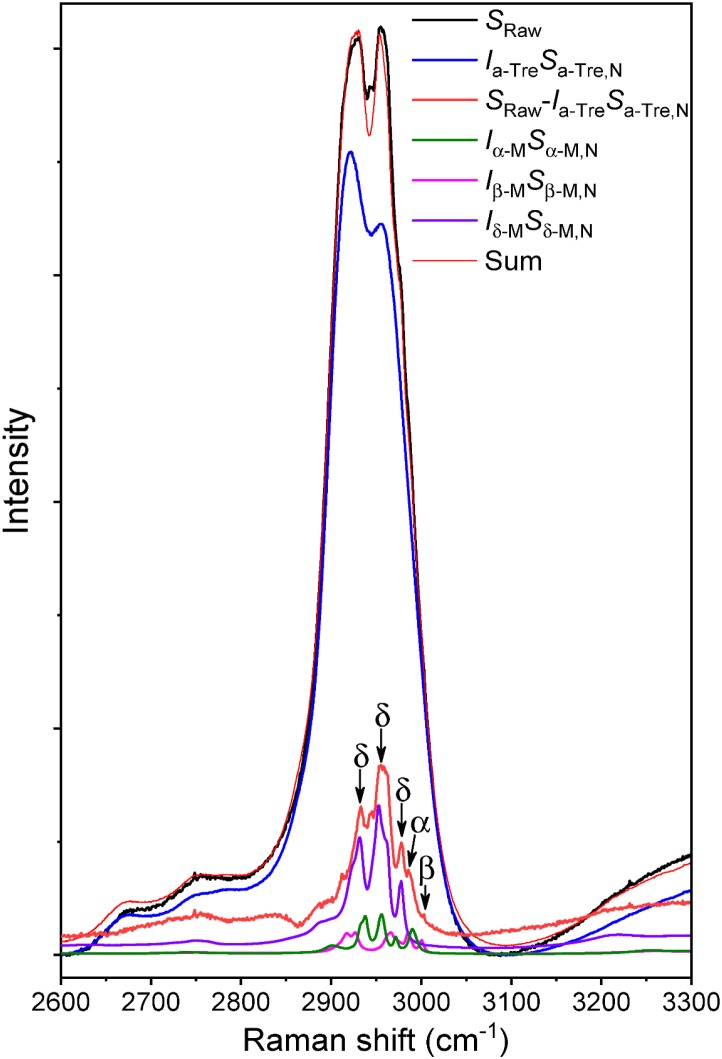
Deconvoluted Raman spectrum of ASFD powder with trehalose and mannitol at a mass ratio of 70:30. Spectral contributions of amorphous trehalose and three mannitol polymorphs (α, δ, β) are detected and separated from the raw mixture spectrum, leading to the sum of the deconvoluted components superimposed well with the measured spectrum.

**FIGURE 6:**
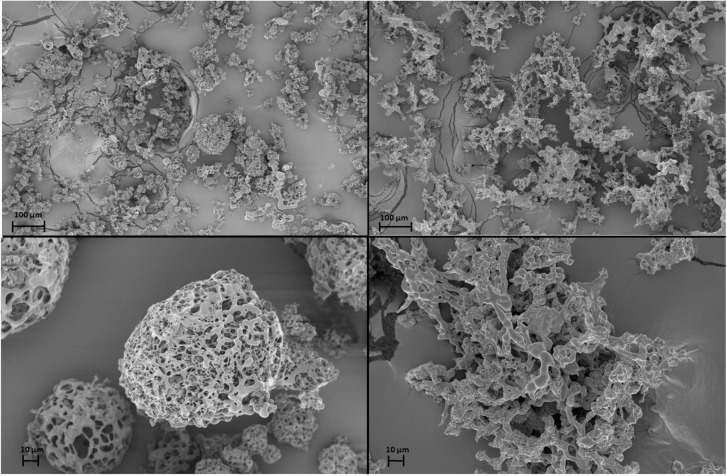
Scanning electron microscope (SEM) images of phage powders. Powder A, Trehalose powder, on the left panels (top and bottom). Powder B, Trehalose-Mannitol powder, on the right panels (top and bottom).

The Raman spectra of the phage powders were obtained in order to determine the solid phase properties of the components in each powder. Powder A consisted of only trehalose and it remained completely amorphous. In powder B, a mixture of trehalose and mannitol, the trehalose remained amorphous, while the mannitol crystallized. The deconvoluted spectrum of powder B is shown in Figure 5. The spectral range for C–H bond stretching (2800–3000 cm^-1^) was used for the deconvolution process. Amorphous trehalose, *I_a-Tre_S_a-Tre,N_*, was identified as the most dominant component in the raw spectrum for its large number of C–H bonds in its molecular structure compared to mannitol. Amorphous trehalose was therefore first subtracted from the raw spectrum, leaving the residual trace for mannitol component as *S_Raw_ - I_a-Tre_S_a-Tre,N_*, in which different polymorphs of mannitol, α, β, and δ, were detected. Characteristic peaks of different mannitol polymorphs used for the deconvolution are marked in Figure 6. A summed spectrum of all the deconvoluted components compared with the raw spectrum shows a good agreement, indicating that all components have been identified and their fractions properly quantified. The obtained intensity factors for each mannitol polymorph, *I_α-M_,I_β-M_*, and *I_δ-M_*, were used directly to calculate the relative amount of each mannitol polymorph in powder B as *I_α-M_/ (I_α-M_ + I_β-M_ + I_δ-M_), I_β-M_/ (I_α-M_ + I_β-M_ + I_δ-M_, and I_δ-M_/ (I_α-M_ + I_β-M_ + I_δ-M_)*, respectively, as the quantification results shown in Table 2. Field Emission SEM images of the dried powders are shown in Figure 6. The images of powder A with 100% trehalose are shown in the left panels. The images of powder B, mixture of trehalose and mannitol solution, are shown in the right panels.

**Table 2:** Crystal structures of sugars in phage powder.

Sample	Trehalose	Mannitol		
		
		α	β	δ
Powder A	amorphous	–	–	–
Powder A2 (Replicate)	amorphous	–	–	–
Powder B	amorphous	15.4 ± 8.0%	15.4 ± 8.0%	69.2 ± 12.0%
Powder B2 (Replicate)	amorphous	19.1 ± 6.8%	10.6 ± 6.5%	70.2 ± 12.2%


## Discussion

Figure 3 shows that ASFD of phage D29 using trehalose and mannitol as excipients provides reasonable biological preservation of the phages during processing. Trehalose is a common disaccharide used for protein preservation since it has a high glass transition temperature of 115°C and is chemically inert (Chen et al., 2000; Wang, 2000). When a protein is desiccated with amorphous trehalose as an excipient, trehalose is believed to immobilize the protein in a glassy matrix, hindering denaturization of the protein; this is also known as the vitrification theory (Jain and Roy, 2010; Mensink et al., 2017). The hydrogen in water molecules is generally assumed to form bonds with the protein to maintain its secondary structure, which can be affected by the removal of water. Trehalose may act as a replacement to water and create hydrogen-bonds with the protein to confer stability (Jain and Roy, 2010; Mensink et al., 2017). Trehalose also has a high affinity to water and can act as a lyoprotectant. Water that is in contact with the trehalose bonded to the phage proteins may be forced into a conformation that prevents or delays ice formation (Jain and Roy, 2010), thereby inhibiting direct contact between the ice crystals and the phage particles. Recent studies have been conducted by Jain and Roy on the effectiveness of trehalose for preserving different types of proteins (Jain and Roy, 2010). In general, protein preservation requires a disaccharide; two other disaccharides that were considered but not used in this experiment was lactose and sucrose. Both lactose and sucrose have low glass transition temperature and will be more prone to crystallization upon room temperature storage. Additionally, sugars like lactose participate in Maillard reaction during gravimetric drying, whereas trehalose does not. The SEM images of powder A showed that the powder particles are porous and spherical in shape. The porous structure is formed when the solution freezes and ice crystals are formed that separate from the freeze concentrate. Many of the powder particles agglomerate. From the low magnification image (Figure 6, top left), it can be seen that the particles vary in size between 5 and 50 μm, which is attributed to the twin fluid atomizer that produces a polydisperse spray in this size range.

Mannitol is a commonly used excipient in tray lyophilization. Mannitol, as a crystalline bulking agent, can prevent the collapse of a powder cake, which aids in drying at higher temperatures and acts as an excipient in preventing trehalose from crystallizing (Pyne et al., 2002; Krauss et al., 2013). The effects of mannitol in the powder are seen from the comparison of the SEM images in Figure 6. The SEM images of powder B (right panels) showed that the particles are porous but lack spherical structure and are larger compared to powder A (left panels). Although particles of both powders have a highly porous structure, the exclusion of mannitol in powder A leads to particles with rounder edges, which is a sign of amorphous trehalose mobility. It is hypothesized that the addition of mannitol limits trehalose mobility by creating a crystalline structure that allows the powder to be dried at higher temperature and larger flow rates.

The solid phase structure is an important powder characteristic as it indicates whether the powder matrix has the necessary stability to protect the phages during storage (Izutsu et al., 1993). Trehalose needs to remain in the form of an amorphous glass to protect the phages from stress during freezing and drying. Mannitol is expected to crystallize during the drying stage and form a supporting skeleton for the particles to prevent further mobility of the trehalose particle structure when drying at higher temperatures. The propensity of mannitol to crystallize during normal spray drying has been studied by Chan et al. (2004) in the case of spray-dried binary mixtures of mannitol and salmon calcitonin. Crystallized mannitol with a combination of different polymorphs were detected in formulations with high mannitol concentrations (>50%), and polymorphic transformations were reported during storage upon exposure to moisture. In comparison, the ASFD freezing step involves a fast cooling process in which mannitol will likely precipitate in amorphous and hemihydrate forms first and subsequently transition to δ-mannitol polymorphs throughout the drying step (Cao et al., 2013). Kim et al. (1998) showed that a faster cooling rate of mannitol generally created more δ-polymorphs of mannitol, which agrees with the results from solid state analysis by Raman spectroscopy (Su et al., 2017). Although δ-mannitol has lower stability than the commonly found β-polymorph, it has been reported to be stable for more than 3 years if stored at room temperatures (25°C) and low humidity (Burger et al., 2000; Chan et al., 2004). The deconvoluted Raman spectra of powder B show that the trehalose remains in the amorphous state which stabilizes the protein.

In the present study, a formulation with 7:3 trehalose to mannitol mass ratio (powder B) was found to provide the highest viability in the resultant dry powder after processing by AFSD. Because trehalose has a glass transition temperature of 16°C at a moisture content of 9.6% (Chen et al., 2000), powder A (100% trehalose) is prone to crystallizing during storage at low temperatures should the moisture content remain constant. Crystallization of trehalose upon storage has been shown to reduce its cryoprotectant properties (Izutsu et al., 1993). The decrease in moisture content seen in Figure 4 for powders A2 and B2 increases the powder’s glass transition temperature to 50°C at a moisture content of 4.9% (Chen et al., 2000), making the powder less prone to crystallization upon storage. Puapermpoonsiri et al. (2010) showed that in their freeze-dried cakes containing phages, the optimal moisture content was in the range of 4–6% w/w, which is shown in the shaded region of Figure 4. Although advanced moisture measurement techniques like Karl Fischer titration would provide better moisture measurements, it was not available for this study.

The results of titer measurements are consistent with other freeze-drying studies that show that, at a proper ratio, trehalose and mannitol are suitable for lyoprotection and cryoprotection of phages (Leung et al., 2016; Zhang et al., 2018). Formulation for powder B had a titer loss of less than 1 log which is an acceptable process loss for phages (Hoe et al., 2013). This demonstrates that ASFD is a viable option for stabilizing biological material and warrants further testing with other biologics. Other processes like conventional freeze-drying and spray drying on preserving phages have been studied elsewhere (Puapermpoonsiri et al., 2010; Leung et al., 2016; Zhang et al., 2018), and further studies comparing ASFD to these techniques will be an interesting topic of further research. The differences in titer loss between powder A and A2 is 2.5 logs whereas the difference between powder B and B2 is 0.2 logs. It is hypothesized that this discrepancy between the original and duplicate run is caused by the additional drying time in the latter. In order to lower the moisture content of the powder to an acceptable range, the drying time was increased, and the moisture content of powder A was approximately halved.

The processing time for ASFD used in this study was approximately 7 h to freeze-dry 10 mL of 100 mg/mL trehalose solution at 10^9^ pfu/mL of phages. A typical freeze-drying process time for tray lyophilization of a similar trehalose solution is approximately 3–5 days (Lu and Pikal, 2004; Leung et al., 2016). The difference in drying time can be attributed to the different drying mechanisms of each process. In ASFD, the solution is frozen nearly instantly upon spraying into the ASFD chamber. The quick freezing rate of the solution causes an even distribution of ice and freeze concentrate throughout the powder, therefore creating smaller ice crystals as compared to were a slower freezing rate used (Mumenthaler and Leuenberger, 1991). Both the water in the freeze concentrate and in the ice is removed via convection by the drying gas. As the ice mass on the particle surface decreases, the gas flow will have lower resistance to subliming the ice in the center of large particles (Mumenthaler and Leuenberger, 1991; Wang et al., 2006). The drying mechanism creates a large specific surface area and particles that are porous without additional processes. In ASFD, the drying mechanism is dependent on the gas flow diffusing water in both the freeze concentrate and the ice out of the powder; therefore, processing time can vary depending on the drying gas flow rate.

There are several limitations to the phage D29 ASFD study. The freeze-drying experiment of phage D29 is limited to only using two excipients in three variations of the formulation. Although this experiment shows the feasibility of using a combination of trehalose and mannitol, a range between the chosen excipients mass ratio could have optimal titer reduction. Conventional freeze-drying of phages has been studied by many previous authors (Puapermpoonsiri et al., 2010; Zhang et al., 2018), while ASFD of phages has not previously been examined; therefore, this study focused solely on ASFD did not compare ASFD with other drying methods. This study showed that phage powders capable of subsequent reconstitution could be produced. Upon reconstitution, the liquid phage feedstock can be applied through nebulization (Carrigy et al., 2017). The phage powders, in its current state, is not produced for inhalation. The phages were stored at room temperature in a drybox, but no long-term storage stability tests have been performed at this time and is in consideration for future studies. At the present research stage of this work, there have not been any plans to scale up the ASFD apparatus and but ASFD is being developed commercially elsewhere (Gladden et al., 2012).

## Conclusion

Results from the present freeze-drying experiments show that ASFD is a feasible method for preserving phage D29. Phages were stabilized with a mixture of trehalose-mannitol and freeze-dried in an ASFD apparatus with acceptable process losses. The preservation of phage D29 in this work indicates that the AFSD process is a promising candidate for powder production with phages that are sensitive to environmental conditions or have a tendency to deactivate upon processing. The phage powders produced in ASFD show one possible formulation for stabilizing phage D29 in a solid form; but phage stabilization is dependent on other factors such as the feed stock solution, processing method, and type of phage. ASFD provided similar biological preservation to traditional methods, but in a shorter processing time than conventional freeze-drying, making ASFD an attractive alternative method for preserving complex biological materials such as phages.

## Data Availability

The datasets generated for this study are available on request to the corresponding author.

## Author Contributions

AL modified and operated the new ASFD apparatus and performed freeze-drying experiments on the phage sugar solutions. AL logged and analyzed the experimental data and drafted the majority of the manuscript and generated the figures in the manuscript. NC contributed with the experimental design, phage formulation suggestions and assisted in the data analysis in microscope images. HW contributed with the Raman spectroscopy, assisted in drafting parts of the “Raman Spectroscopy” section, and edited “Solid Phase Properties” section. MH contributed in performing plaque assays to determine the titer of the phages. DS contributed by providing phage D29 for the experiments and editorial suggestions in the manuscript. AM contributed as a co-supervisor and gave suggestions in both experimental work and drafting of the manuscript. RV contributed with providing suggestions in analysis work which include Raman spectroscopy and titer measurements and provided editorial suggestions in the manuscript. WF contributed as the supervisor of this project – encouraged and guided both experimental work and drafting of the manuscript.

## Conflict of Interest Statement

The authors declare that the research was conducted in the absence of any commercial or financial relationships that could be construed as a potential conflict of interest.
